# Supplementary figures and images for: Mettl3-mediated m6A modification of Fgf16 restricts cardiomyocyte proliferation during heart regeneration
Source: eLife. 2022 Nov 18;11:e77014. doi: 10.7554/eLife.77014 (PMC9674341; doi:10.7554/eLife.77014)

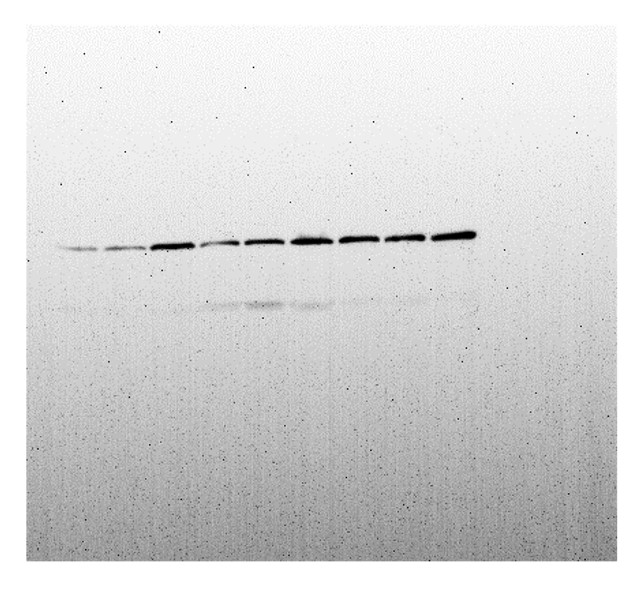

Supplement: Figure 1—source data 1. [file elife-77014-fig1-data1.zip › Figure 1-source data 1.jpg]

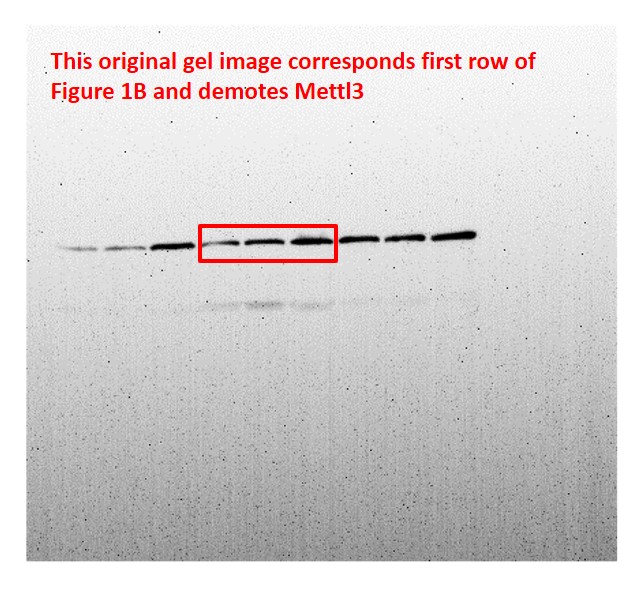

Supplement: Figure 1—source data 1. [file elife-77014-fig1-data1.zip › Figure 1-source data 1-bands.jpg]

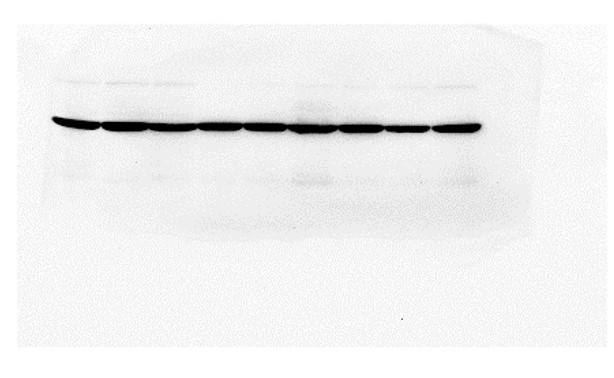

Supplement: Figure 1—source data 2. [file elife-77014-fig1-data2.zip › Figure 1-source data 2.jpg]

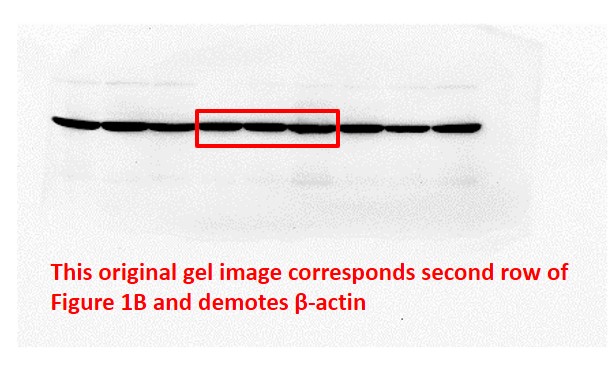

Supplement: Figure 1—source data 2. [file elife-77014-fig1-data2.zip › Figure 1-source data 2-bands.jpg]

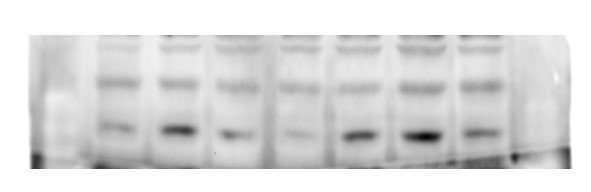

Supplement: Figure 1—source data 3. [file elife-77014-fig1-data3.zip › Figure 1-source data 3.jpg]

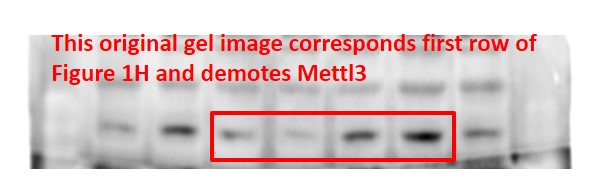

Supplement: Figure 1—source data 3. [file elife-77014-fig1-data3.zip › Figure 1-source data 3-bands.jpg]

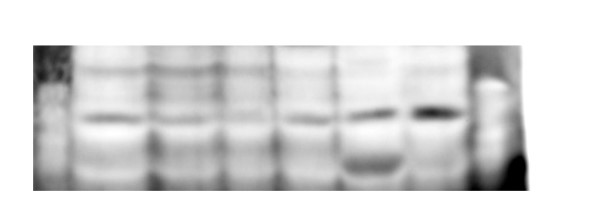

Supplement: Figure 1—source data 4. [file elife-77014-fig1-data4.zip › Figure 1-source data 4.jpg]

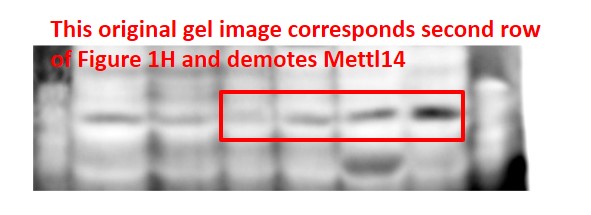

Supplement: Figure 1—source data 4. [file elife-77014-fig1-data4.zip › Figure 1-source data 4-bands.jpg]

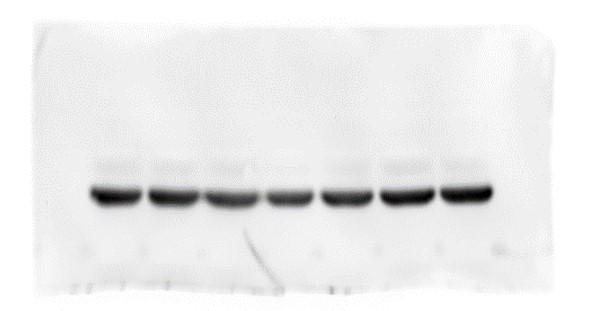

Supplement: Figure 1—source data 5. [file elife-77014-fig1-data5.zip › Figure 1-source data 5.jpg]

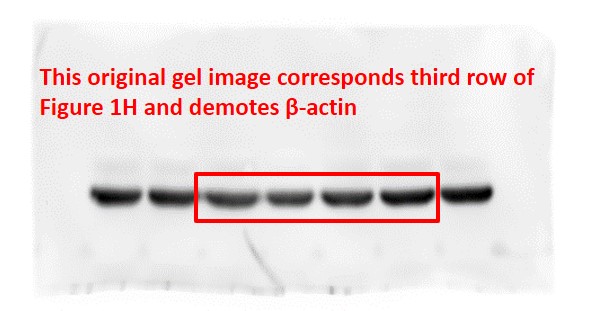

Supplement: Figure 1—source data 5. [file elife-77014-fig1-data5.zip › Figure 1-source data 5-bands.jpg]

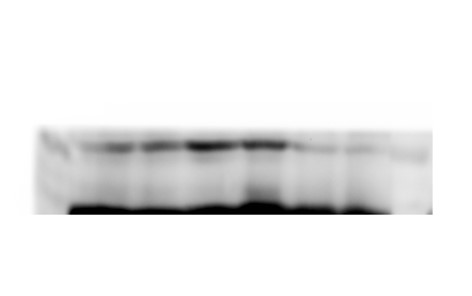

Supplement: Figure 1—figure supplement 1—source data 1. [file elife-77014-fig1-figsupp1-data1.zip › Figure 1-figure supplement 1-source data 1.jpg]

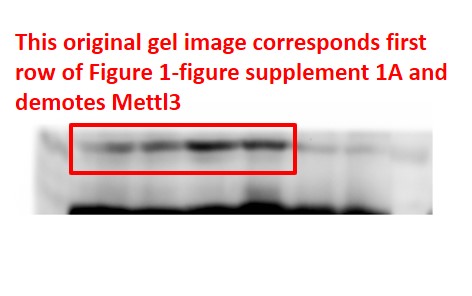

Supplement: Figure 1—figure supplement 1—source data 1. [file elife-77014-fig1-figsupp1-data1.zip › Figure 1-figure supplement 1-source data 1-bands.jpg]

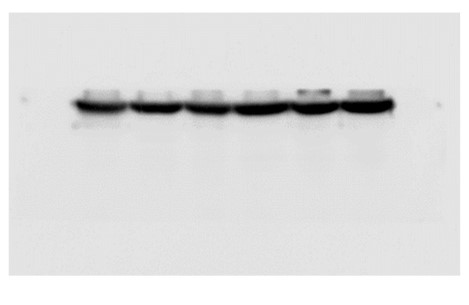

Supplement: Figure 1—figure supplement 1—source data 2. [file elife-77014-fig1-figsupp1-data2.zip › Figure 1-figure supplement 1-source data 2.jpg]

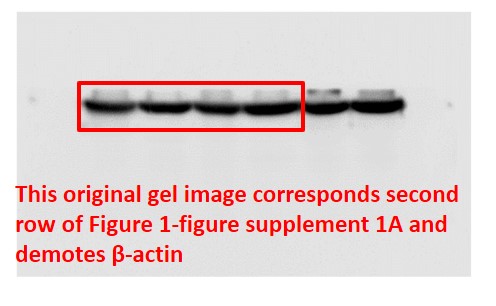

Supplement: Figure 1—figure supplement 1—source data 2. [file elife-77014-fig1-figsupp1-data2.zip › Figure 1-figure supplement 1-source data 2-bands.jpg]

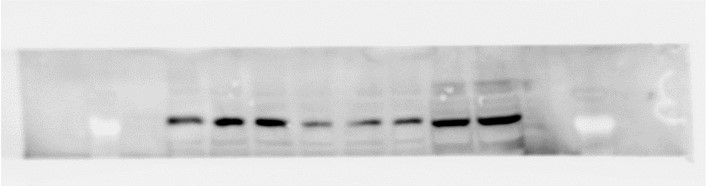

Supplement: Figure 2—source data 1. [file elife-77014-fig2-data1.zip › Figure 2-source data 1.jpg]

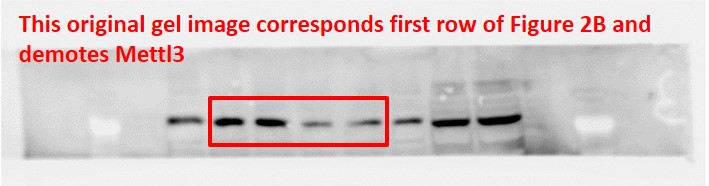

Supplement: Figure 2—source data 1. [file elife-77014-fig2-data1.zip › Figure 2-source data 1-bands.jpg]

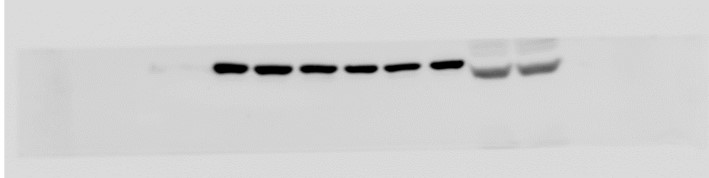

Supplement: Figure 2—source data 2. [file elife-77014-fig2-data2.zip › Figure 2-source data 2.jpg]

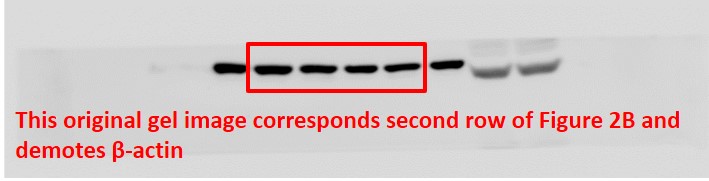

Supplement: Figure 2—source data 2. [file elife-77014-fig2-data2.zip › Figure 2-source data 2-bands.jpg]

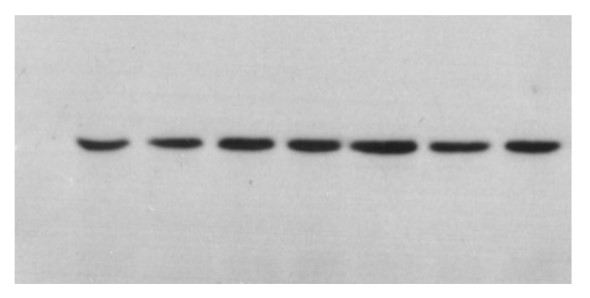

Supplement: Figure 2—source data 3. [file elife-77014-fig2-data3.zip › Figure 2-source data 3.jpg]

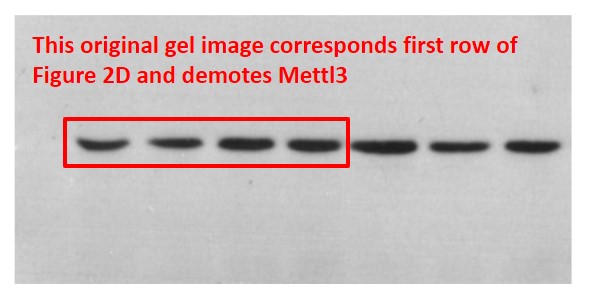

Supplement: Figure 2—source data 3. [file elife-77014-fig2-data3.zip › Figure 2-source data 3-bands.jpg]

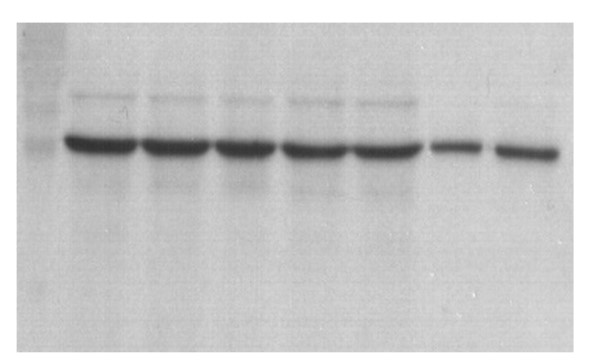

Supplement: Figure 2—source data 4. [file elife-77014-fig2-data4.zip › Figure 2-source data 4.jpg]

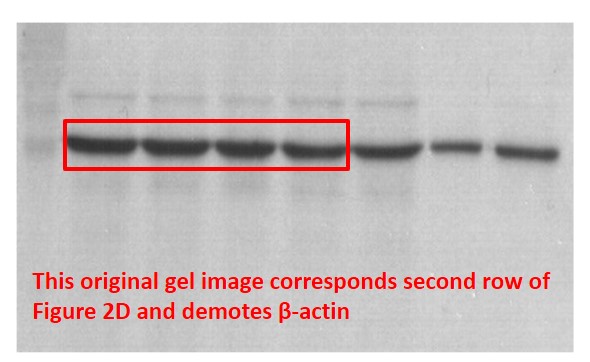

Supplement: Figure 2—source data 4. [file elife-77014-fig2-data4.zip › Figure 2-source data 4-bands.jpg]

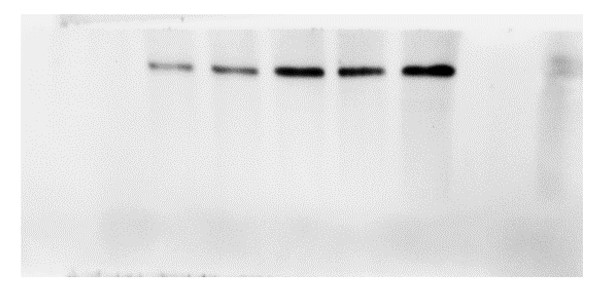

Supplement: Figure 2—figure supplement 1—source data 1. [file elife-77014-fig2-figsupp1-data1.zip › Figure 2-figure supplement 1-source data 1.jpg]

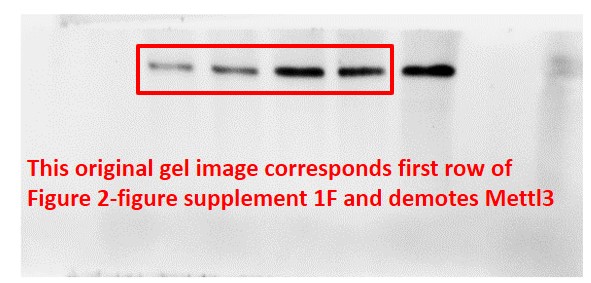

Supplement: Figure 2—figure supplement 1—source data 1. [file elife-77014-fig2-figsupp1-data1.zip › Figure 2-figure supplement 1-source data 1-bands.jpg]

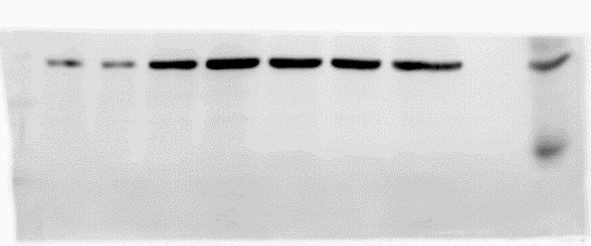

Supplement: Figure 2—figure supplement 1—source data 2. [file elife-77014-fig2-figsupp1-data2.zip › Figure 2-figure supplement 1-source data 2.jpg]

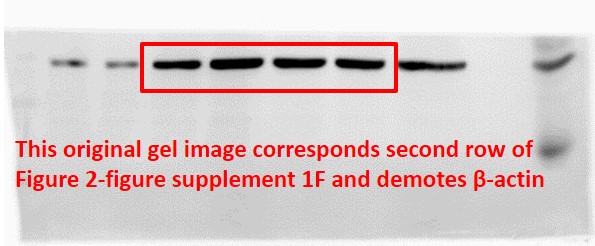

Supplement: Figure 2—figure supplement 1—source data 2. [file elife-77014-fig2-figsupp1-data2.zip › Figure 2-figure supplement 1-source data 2-bands.jpg]

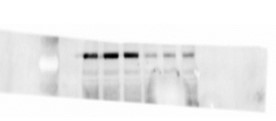

Supplement: Figure 3—source data 1. [file elife-77014-fig3-data1.zip › Figure 3-source data 1.jpg]

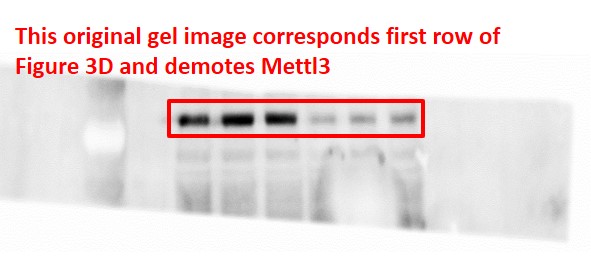

Supplement: Figure 3—source data 1. [file elife-77014-fig3-data1.zip › Figure 3-source data 1-bands.jpg]

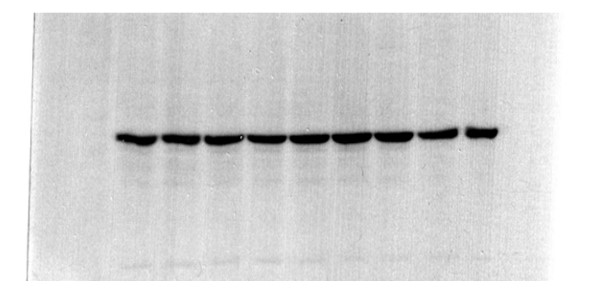

Supplement: Figure 3—source data 2. [file elife-77014-fig3-data2.zip › Figure 3-source data 2.jpg]

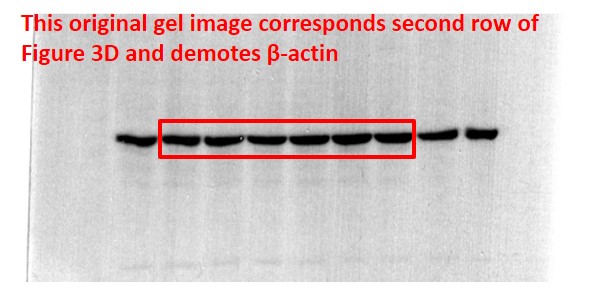

Supplement: Figure 3—source data 2. [file elife-77014-fig3-data2.zip › Figure 3-source data 2-bands.jpg]

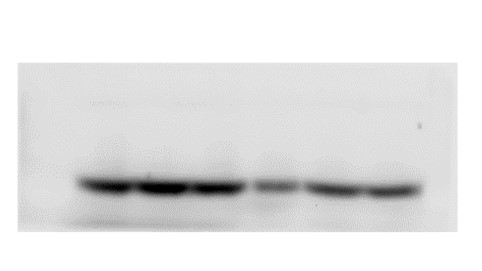

Supplement: Figure 4—figure supplement 2—source data 1. [file elife-77014-fig4-figsupp2-data1.zip › Figure 4-figure supplement 2-source data 1.jpg]

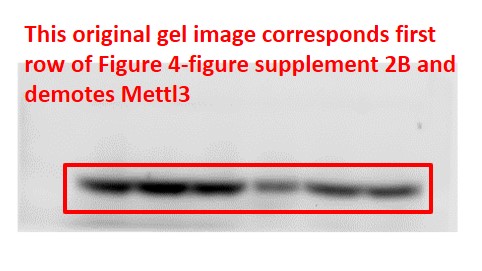

Supplement: Figure 4—figure supplement 2—source data 1. [file elife-77014-fig4-figsupp2-data1.zip › Figure 4-figure supplement 2-source data 1-bands.jpg]

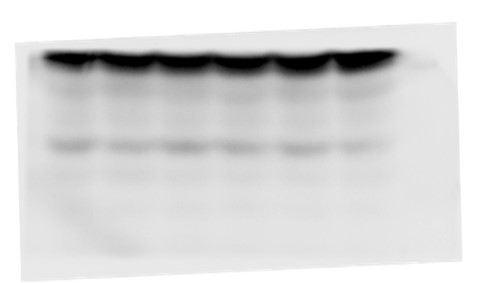

Supplement: Figure 4—figure supplement 2—source data 2. [file elife-77014-fig4-figsupp2-data2.zip › Figure 4-figure supplement 2-source data 2.jpg]

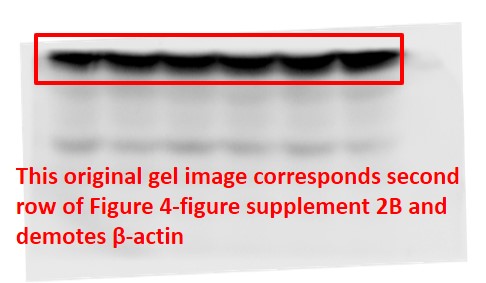

Supplement: Figure 4—figure supplement 2—source data 2. [file elife-77014-fig4-figsupp2-data2.zip › Figure 4-figure supplement 2-source data 2-bands.jpg]

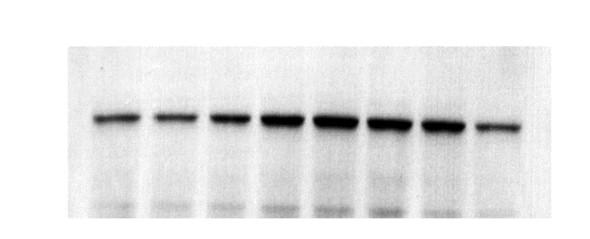

Supplement: Figure 5—source data 1. [file elife-77014-fig5-data1.zip › Figure 5-source data 1.jpg]

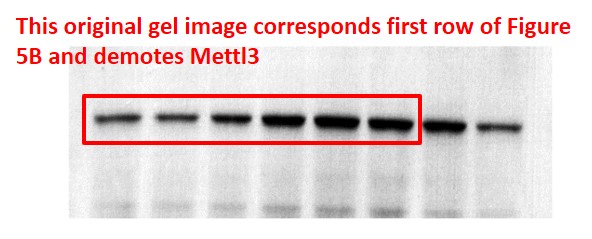

Supplement: Figure 5—source data 1. [file elife-77014-fig5-data1.zip › Figure 5-source data 1-bands.jpg]

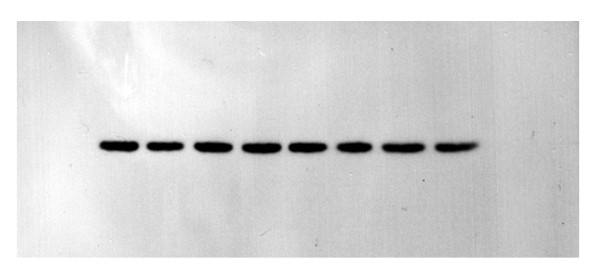

Supplement: Figure 5—source data 2. [file elife-77014-fig5-data2.zip › Figure 5-source data 2.jpg]

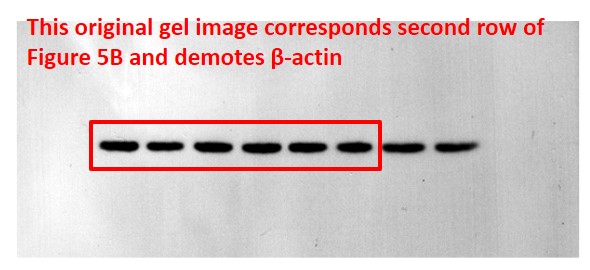

Supplement: Figure 5—source data 2. [file elife-77014-fig5-data2.zip › Figure 5-source data 2-bands.jpg]

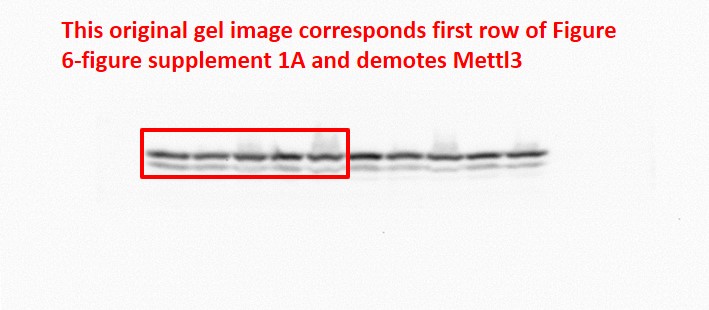

Supplement: Figure 6—figure supplement 1—source data 1. [file elife-77014-fig6-figsupp1-data1.zip › Figure 6-figure supplement 1-source data 1-bands.jpg]

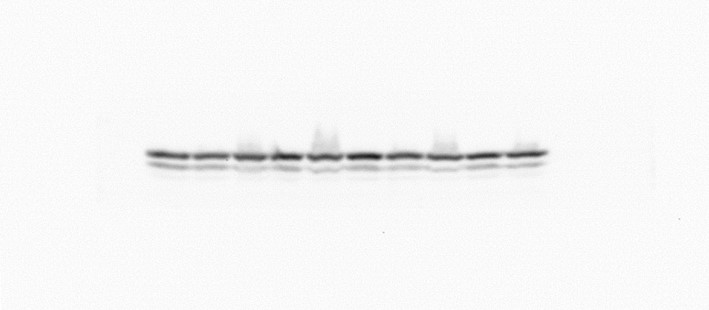

Supplement: Figure 6—figure supplement 1—source data 1. [file elife-77014-fig6-figsupp1-data1.zip › Figure 6-figure supplement 1-source data 1.jpg]

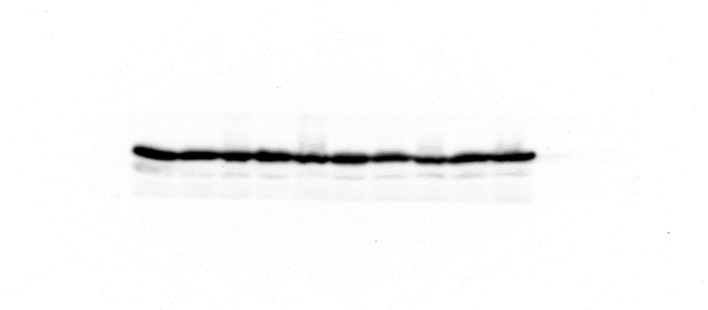

Supplement: Figure 6—figure supplement 1—source data 2. [file elife-77014-fig6-figsupp1-data2.zip › Figure 6-figure supplement 1-source data 2.jpg]

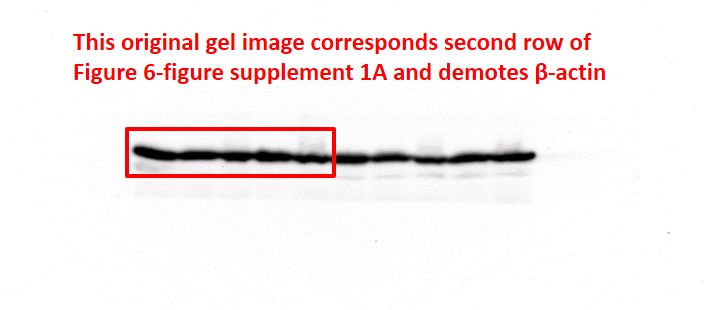

Supplement: Figure 6—figure supplement 1—source data 2. [file elife-77014-fig6-figsupp1-data2.zip › Figure 6-figure supplement 1-source data 2-bands.jpg]

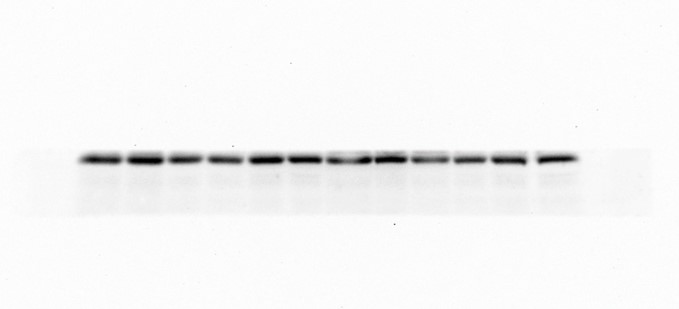

Supplement: Figure 6—figure supplement 1—source data 3. [file elife-77014-fig6-figsupp1-data3.zip › Figure 6-figure supplement 1-source data 3.jpg]

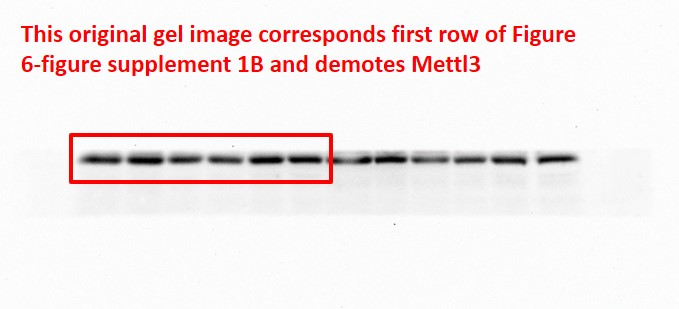

Supplement: Figure 6—figure supplement 1—source data 3. [file elife-77014-fig6-figsupp1-data3.zip › Figure 6-figure supplement 1-source data 3-bands.jpg]

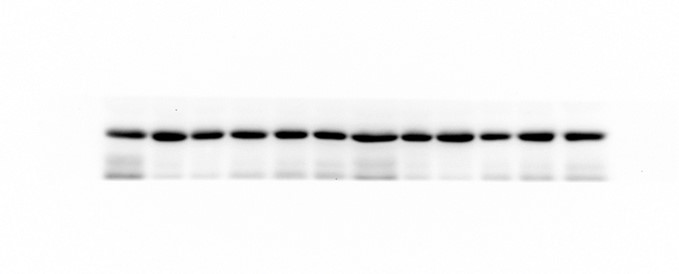

Supplement: Figure 6—figure supplement 1—source data 4. [file elife-77014-fig6-figsupp1-data4.zip › Figure 6-figure supplement 1-source data 4.jpg]

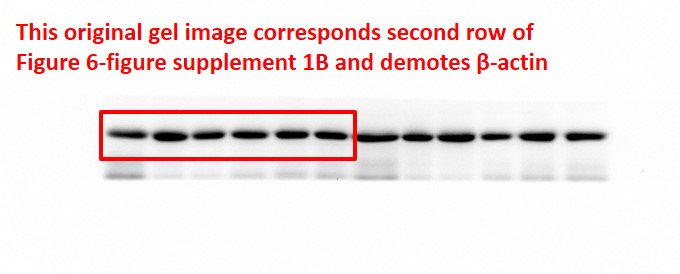

Supplement: Figure 6—figure supplement 1—source data 4. [file elife-77014-fig6-figsupp1-data4.zip › Figure 6-figure supplement 1-source data 4-bands.jpg]

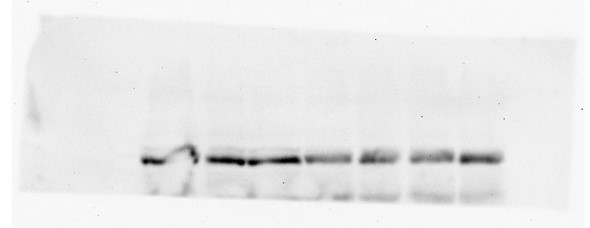

Supplement: Figure 8—source data 1. [file elife-77014-fig8-data1.zip › Figure 8-source data 1.jpg]

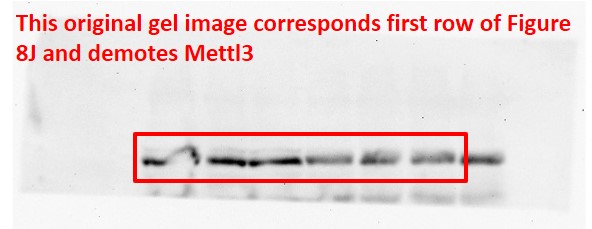

Supplement: Figure 8—source data 1. [file elife-77014-fig8-data1.zip › Figure 8-source data 1-bands.jpg]

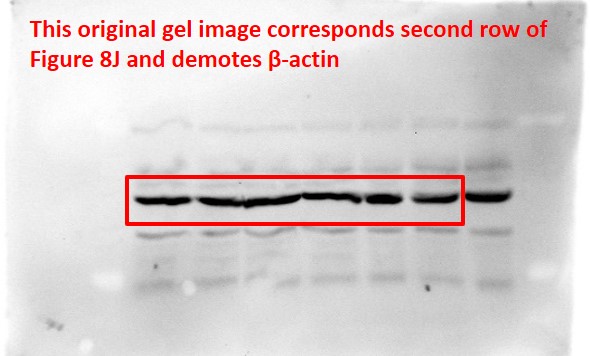

Supplement: Figure 8—source data 2. [file elife-77014-fig8-data2.zip › Figure 8-source data 2-bands.jpg]

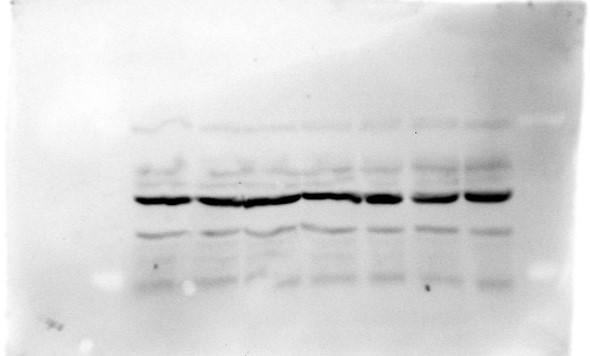

Supplement: Figure 8—source data 2. [file elife-77014-fig8-data2.zip › Figure 8-source data 2.jpg]

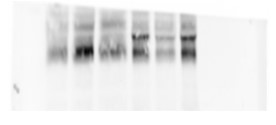

Supplement: Figure 8—source data 3. [file elife-77014-fig8-data3.zip › Figure 8-source data 3.jpg]

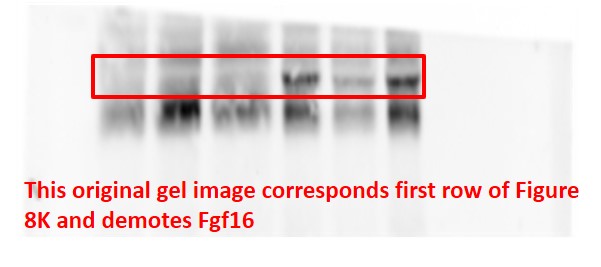

Supplement: Figure 8—source data 3. [file elife-77014-fig8-data3.zip › Figure 8-source data 3-bands.jpg]

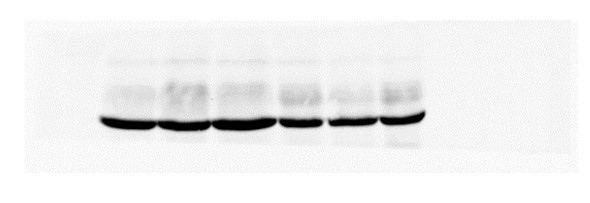

Supplement: Figure 8—source data 4. [file elife-77014-fig8-data4.zip › Figure 8-source data 4.jpg]

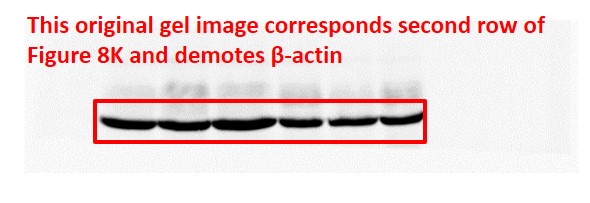

Supplement: Figure 8—source data 4. [file elife-77014-fig8-data4.zip › Figure 8-source data 4-bands.jpg]

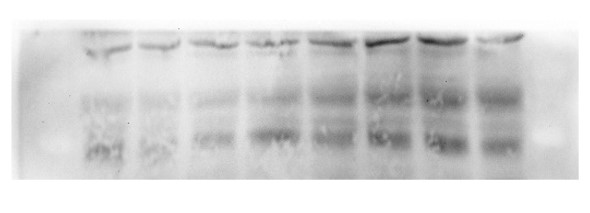

Supplement: Figure 8—source data 5. [file elife-77014-fig8-data5.zip › Figure 8-source data 5.jpg]

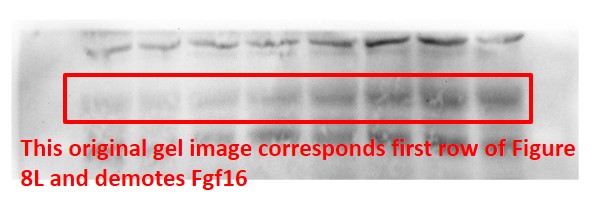

Supplement: Figure 8—source data 5. [file elife-77014-fig8-data5.zip › Figure 8-source data 5-bands.jpg]

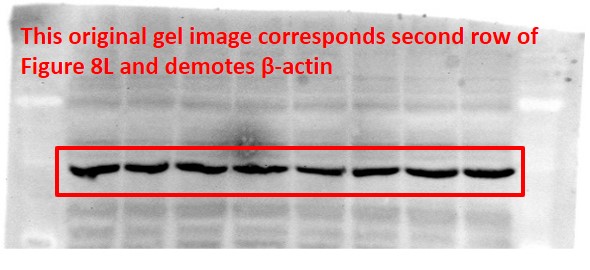

Supplement: Figure 8—source data 6. [file elife-77014-fig8-data6.zip › Figure 8-source data 6-bands.jpg]

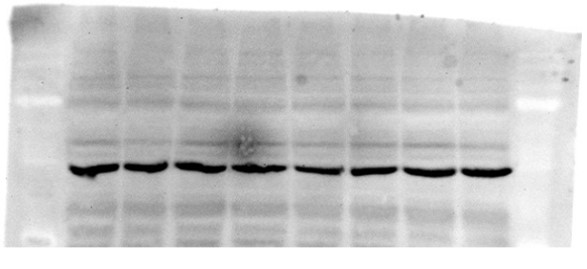

Supplement: Figure 8—source data 6. [file elife-77014-fig8-data6.zip › Figure 8-source data 6.jpg]

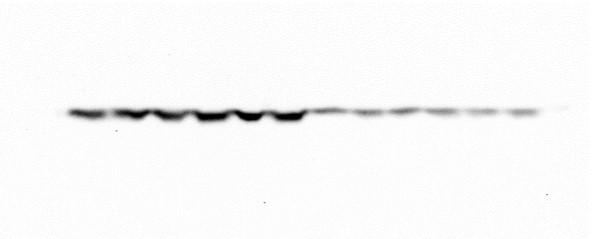

Supplement: Figure 8—source data 7. [file elife-77014-fig8-data7.zip › Figure 8-source data 7.jpg]

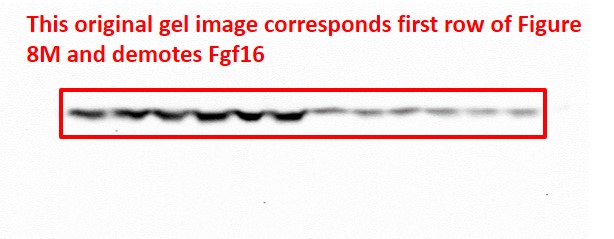

Supplement: Figure 8—source data 7. [file elife-77014-fig8-data7.zip › Figure 8-source data 7-bands.jpg]

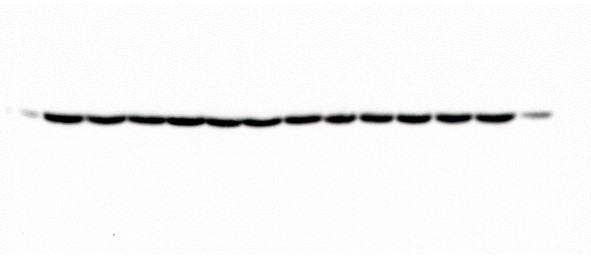

Supplement: Figure 8—source data 8. [file elife-77014-fig8-data8.zip › Figure 8-source data 8.jpg]

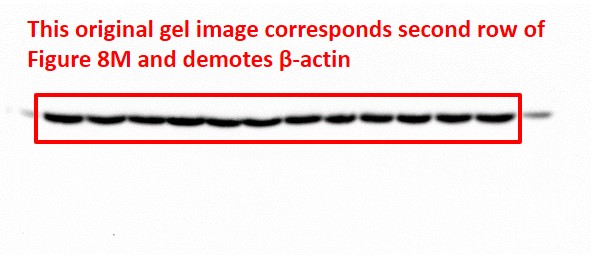

Supplement: Figure 8—source data 8. [file elife-77014-fig8-data8.zip › Figure 8-source data 8-bands.jpg]

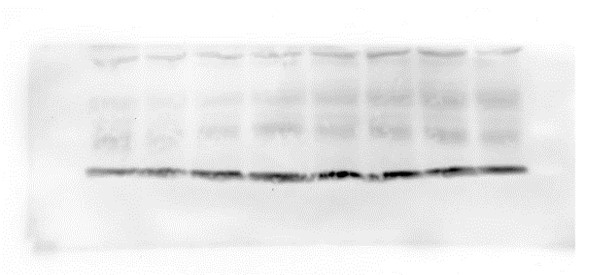

Supplement: Figure 9—source data 1. [file elife-77014-fig9-data1.zip › Figure 9-source data 1.jpg]

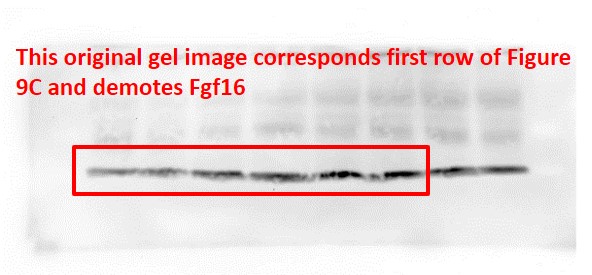

Supplement: Figure 9—source data 1. [file elife-77014-fig9-data1.zip › Figure 9-source data 1-bands.jpg]

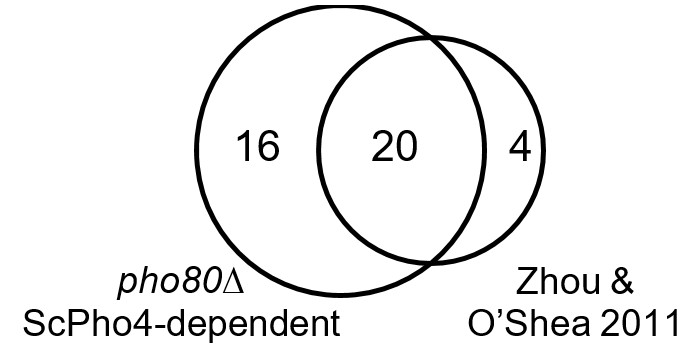

Supplement: Figure 9—source data 1. [file elife-77014-fig9-data1.zip › elife-25157-resp-fig1-v2.jpg]

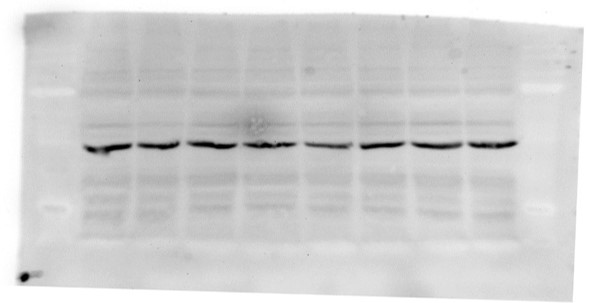

Supplement: Figure 9—source data 2. [file elife-77014-fig9-data2.zip › Figure 9-source data 2.jpg]

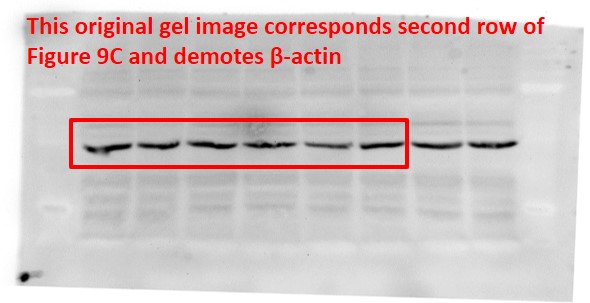

Supplement: Figure 9—source data 2. [file elife-77014-fig9-data2.zip › Figure 9-source data 2-bands.jpg]
