# Supplementary material for: Mettl3-mediated m6A modification of Fgf16 restricts cardiomyocyte proliferation during heart regeneration
Source: eLife. 2022 Nov 18;11:e77014. doi: 10.7554/eLife.77014 (PMC9674341; doi:10.7554/eLife.77014)
Supplement: Supplementary file 1. [file elife-77014-supp1.docx]

**Supplementary file 1. Sequences of siRNAs used in this study.**

| **Species** | **Target gene** | **NCBI Reference No.** | **siRNAs** | |
| --- | --- | --- | --- | --- |
|  |  |  | **Name** | **Sequence (5’-3’)** |
| Mouse | *Mettl3* | NM_019721.2 | siMettl3 | GCACACTGATGAATCTTTA |
|  | *Fgf16* | NM_030614.2 | siFgf16 | GCTCTATGGATCGAAGAAACT |
|  | *Ythdf1* | NM_173761.3 | siYthdf1 | GACAGTCCAATCCGAGTAACA |
|  | *Ythdf2* | NM_145393.4 | siYthdf2 | GCAGCACAGAGCATGGTAACA |
|  | *Mettl14* | NM_201638.2 | siMettl14 | GCATTGGTGCTGTGTTAAA3 |
|  | *Fto* | NM_011936.2 | siFto | GAGTGCTCAACAGGCACCTTGGATT |
| H9c2 cells | *Mettl3* | NM_001024794.1 | siMettl3 | GCACACTGATGAATCTTTA |
|  | *Fgf16* | NM_021867.3 | siFgf16 | GACTCGGAGAGACAGTATTAT |
|  | *Mis12* | NM_001047972.1 | siMis12 | GGCAGGTATCAAGGAACTA |
|  | *Six5* | NM_001372077.1 | siSix5 | GGAGAGGAAATCCCAGGAA |
|  | *Ythdf2* | NM_001047099.1 | siYthdf2 | GGTTCTGGATCTACTCCTT |
| Negative control (NC) | | | siNC | GTCCAGATTGTCCGCAACTA |
