# Supplementary material for: Mettl3-mediated m6A modification of Fgf16 restricts cardiomyocyte proliferation during heart regeneration
Source: eLife. 2022 Nov 18;11:e77014. doi: 10.7554/eLife.77014 (PMC9674341; doi:10.7554/eLife.77014)
Supplement: Supplementary file 2. [file elife-77014-supp2.docx]

**Supplementary file 2. Primer sequences for real-time PCR analysis in mouse.**

| **Gene** | **NCBI Reference No.** | **Primer sequence (5’-3’)** | |
| --- | --- | --- | --- |
|  |  | **Forward primer** | **Reverse primer** |
| ***Mettl3*** | NM_019721.2 | CTGGGCACTTGGATTTAAGGAA | TGAGAGGTGGTGTAGCAACTT |
| ***Mettl14*** | NM_201638.2 | CTCCAAACTCAAAACGGAAGTGT | ATGGGGATTTAAGCTCTGCGT |
| ***Alkbh5*** | NM_172943.4 | CGCGGTCATCAACGACTACC | ATGGGCTTGAACTGGAACTTG |
| ***Fto*** | NM_011936.2 | TTCATGCTGGATGACCTCAATG | GCCAACTGACAGCGTTCTAAG |
| ***Fgf16*** | NM_030614.2 | CCATGACTCAAGGGAGCTTT | CTATGCCCAATCCTGAAGGT |
| ***Ythdf1*** | NM_173761.3 | ACAGTTACCCCTCGATGAGTG | GGTAGTGAGATACGGGATGGGA |
| ***Ythdf2*** | NM_145393.4 | GCTTGCCTGCTACATAGTGAGA | AACTGAACTGCTTAACCTTCTGG |
| ***Pml*** | NM_008884.5 | GAGCAGGTAATCGCCCAACT | GAGCAAAGTCCACCTGGGTT |
| ***Aspm*** | NM_009791.4 | GGCTACTGTGATTCAGGCCA | TGCTTTTGCCTCTGCAGTTC |
| ***Cdc20*** | NM_023223.2 | ATCCAGTGGTTCACGGTCTG | AAGCCCACATACTTCCTGGC |
| ***H2afx*** | NM_010436.2 | TGGTCTCTCAGCGTTGTTCG | CTACAGGGAACTGAAGGCCG |
| ***Mis12*** | NM_025993.3 | CGCCTGTCACATTCCAGTCT | TGTACATGCCACAGTGTACCTT |
| ***Sac3d1*** | NM_133678.3 | TGGATGGACTCCAGGAAGCAC | AGGGTGTGTCCCTGCAACTTATTC |
| ***Pprc1*** | NM_001081214.1 | TGAGGATTTTGGAGAGAGCAGA | TTGAGCAGCTTGTGCAGAGA |
| ***Mif*** | NM_010798.3 | CGCGCTTTGTACCGTCCTC | ACGTGCACTGCGATGTACT |
| ***Mycn*** | NM_008709.3 | AAGTCACCTTGTTCCGGTCC | TTCCCAGGGGCATCAAATGG |
| ***Six5*** | NM_011383.1 | CAGCTTCTCAGCACCCCTAC | CCTGTTGTAGCCCCCAGAAG |
| ***Pdgfb*** | NM_011057.4 | TGTCTGTCCATTGCCTCCAC | CTAGGGCTCTCCCTCTCTCC |
| ***Gapdh*** | NM_001289726.1 | TGTGTCCGTCGTGGATCTGA | TTGCTGTTGAAGTCGCAGGAG |
