# Supplementary material for: Mettl3-mediated m6A modification of Fgf16 restricts cardiomyocyte proliferation during heart regeneration
Source: eLife. 2022 Nov 18;11:e77014. doi: 10.7554/eLife.77014 (PMC9674341; doi:10.7554/eLife.77014)
Supplement: Supplementary file 3. [file elife-77014-supp3.docx]

**Supplementary file 3. Primer sequences for real-time PCR analysis in H9c2 cells.**

| **Gene** | **NCBI Reference No.** | **Primer sequence (5’-3’)** | |
| --- | --- | --- | --- |
|  |  | **Forward primer** | **Reverse primer** |
| ***Mettl3*** | NM_001024794.1 | CTGGGCACTTGGATTTAAGGAA | TGAGAGGTGGTGTAGCAACTT |
| ***Fgf16*** | NM_021867.3 | TGTACCTAGGAATGAATGAGCGAGG | GCCACATAATACTGTCTCTCCGAGT |
| ***Ythdf2*** | NM_001047099.1 | GAGCAGAGACCAAAAGGTCAAG | CTGTGGGCTCAAGTAAGGTTC |
| ***Mis12*** | NM_001047972.1 | AAGTTGGGCTTGTTATACTCCT | AAGTTGGGCTTGTTATACTCCT |
| ***Six5*** | NM_001372077.1 | GGGTGAATATGCCGAGCTCTA | CATCCCAGATGGTCTTGGGC |
| ***Gapdh*** | NM_017008.4 | TGTGTCCGTCGTGGATCTGA | TTGCTGTTGAAGTCGCAGGAG |
