# Supplementary material for: Mettl3-mediated m6A modification of Fgf16 restricts cardiomyocyte proliferation during heart regeneration
Source: eLife. 2022 Nov 18;11:e77014. doi: 10.7554/eLife.77014 (PMC9674341; doi:10.7554/eLife.77014)
Supplement: Supplementary file 4. [file elife-77014-supp4.docx]

**>pGL-RF**

CMV promoter

BamHI

XhoI

ClaI

Gaussia Dura Luc

pTK promoter

XbaI

Red Firefly Luc

bGHpA

actagttattaatagtaatcaattacggggtcattagttcatagcccatatatggagttccgcgttacataacttacggtaaatggcccgcctggctgaccgcccaacgacccccgcccattgacgtcaataatgacgtatgttcccatagtaacgccaatagggactttccattgacgtcaatgggtggagtatttacggtaaactgcccacttggcagtacatcaagtgtatcatatgccaagtacgccccctattgacgtcaatgacggtaaatggcccgcctggcattatgcccagtacatgaccttatgggactttcctacttggcagtacatctacgtattagtcatcgctattaccatggtgatgcggttttggcagtacatcaatgggcgtggatagcggtttgactcacggggatttccaagtctccaccccattgacgtcaatgggagtttgttttggcaccaaaatcaacgggactttccaaaatgtcgtaacaactccgccccattgacgcaaatgggcggtaggcgtgtacggtgggaggtctatataagcagagctctctggctaactagagaacccactgcttactggcttatcgaaattaatacgactcactataggGGATCCgccaccatgggagtcaaagttctgtttgccctgatctgcatcgctgtggccgaggccaagcccaccgagaacaacgaagacttcaacatcgtggccgtggccagcaacttcgcgaccacggatctcgatgctgaccgcgggaagttgcccggcaagaagctgccgctggaggtgctcaaagagttggaagccaatgcccggaaagctggctgcaccaggggctgtctgatctgcctgtcccacatcaagtgcacgcccaagatgaagaagttcatcccaggacgctgccacacctacgaaggcgacaaagagtccgcacagggcggcataggcgaggcgatcgtcgacattcctgagattcctgggttcaaggacttggagcccttggagcagttcatcgcacaggtcgatctgtgtgtggactgcacaactggctgcctcaaagggcttgccaacgtgcagtgttctgacctgctcaagaagtggctgccgcaacgctgtgcgacctttgccagcaagatccagggccaggtggacaagatcaagggggccggtggtgacCTCGAGtaaATCGATtgataagatacattgatgagtttggacaaaccacaactagaatgcagtgaaaaaaatgctttatttgtgaaatttgtgatgctattgctttatttgtaaccattataagctgcaataaacaagcttgttaacaatgagtcttcggacctcgcgggggccgcttaagcggtggttagggtttgtctgacgcggggggagggggaaggaacgaaacactctcattcggaggcggctcggggtttggtcttggtggccacgggcacgcagaagagcgccgcgatcctcttaagcacccccccgccctccgtggaggcgggggtttggtcggcgggtggtaactggcgggccgctgactcgggcgggtcgcgcgccccagagtgtgaccttttcggtctgctcgcagacccccgggcggcgccgccgcggcggcgacgggctcgctgggtcctaggctccatggggaccgtatacgtggacaggctctggagcatccgcacgactgcggtgatattaccggagaccttctgcgggacgagccgggtcacgcggctgacgcggagcgtccgttgggcgacaaacaccaggacggggcacaggtacactatcttgtcacccggaggcgcgagggactgcaggagcttcagggagtggcgcagctgcttcatccccgtggcccgttgctcgcgtttgctggcggtgtccccggaagaaatatatttgcatgtctttagttctatgatgacacaaaccccgcccagcgtcttgtcattggcgaattcgaacacgcagatgcagtcggggcggcgcggtcccaggtccacttcgcatattaaggtgacgcgtgtggcctcgaacaccgagcgaccctgcagcgacccgcttaaTCTAGAgccaccatggaaaatatggaaaacgacgagaacatcgtggtgggccccaagcccttctaccccatcgaggaaggcagcgccggcacccagctgcggaagtacatggaaagatacgccaagctgggcgccattgccttcaccaacgccgtgaccggcgtggactacagctacgccgagtacctggaaaagagctgctgcctgggcaaggctctgcagaactacggcctggtggtggacggccggatcgccctgtgcagcgagaactgcgaggaattcttcatccccgtgatcgccggcctgttcatcggcgtgggcgtggctcccaccaacgagatctacaccctgcgggagctggtgcacagcctgggcatcagcaagcccaccatcgtgttcagcagcaagaagggcctggacaaagtcatcaccgtgcagaaaaccgtgaccaccatcaagaccatcgtgatcctggacagcaaggtggactaccggggctaccagtgcctggacaccttcatcaagcggaacaccccccctggcttccaggccagcagcttcaagaccgtggaggtggaccggaaagaacaggtggccctgatcatgaacagcagcggcagcaccggcctgcccaagggcgtgcagctgacccacgagaacaccgtgacccggttcagccacgccagggaccccatctacggcaaccaggtgtcccccggcaccgccgtgctgaccgtggtgcccttccaccacggcttcggcatgttcaccaccctgggctacctgatctgcggcttccgggtggtgatgctgaccaagttcgacgaggaaaccttcctgaaaaccctgcaggactacaagtgcacctacgtgattctggtgcccaccctgttcgccatcctgaacaagagcgagctgctgaacaagtacgacctgagcaacctggtggagatcgccagcggcggagcccccctgagcaaagaagtgggagaggccgtcgccaggcggttcaatctgcccggcgtgcggcagggctacggcctgaccgagacaaccagcgccatcatcatcacccccgagggcgacgacaagcctggagccagcggcaaggtggtgcccctgttcaaggccaaagtgatcgacctggacaccaagaagagcctgggccccaacagacggggcgaagtgtgcgtgaagggccccatgctgatgaagggctacgtgaacaaccccgaggccaccaaagagctgatcgacgaagagggctggctgcacaccggcgacatcggctactacgacgaagagaagcacttcttcatcgtggaccggctgaagagcctgatcaagtacaagggctatcaggtgccccctgccgagctggaaagcgtcctgctgcagcaccccagcatcttcgacgccggcgtggccggggtgccagatcctgtggccggcgagctgcctggcgccgtggtggtgctggaatccggcaagaacatgaccgagaaagaagtgatggactacgtcgccagccaggtgtccaacgccaagcggctgagaggcggcgtgagattcgtggacgaagtgccaaagggcctgaccggcaagatcgacggcagggccatccgggagatcctgaagaaacccgtggccaagatgtgatgagcggccgcaaaatcagcctcgactgtgccttctagttgccagccatctgttgtttgcccctcccccgtgccttccttgaccctggaaggtgccactcccactgtcctttcctaataaaatgaggaaattgcatcgccggctggatgatcctccagcgcggggatctcatgctggagttcttcgcccaccccaacttgtttattgcagcttataatggttacaaataaagcaatagcatcacaaatttcacaaataaagcatttttttcactgcattctagttgtggtttgtccaaactcatcaatgtatcttatcatgtctgtataccgtcgacctctagctagagcttggcgtaatcatggtcattaccaatgcttaatcagtgaggcacctatctcagcgatctgtctatttcgttcatccatagttgcctgactccccgtcgtgtagataactacgatacgggagggcttaccatctggccccagcgctgcgatgataccgcgagaaccacgctcaccggctccggatttatcagcaataaaccagccagccggaagggccgagcgcagaagtggtcctgcaactttatccgcctccatccagtctattaattgttgccgggaagctagagtaagtagttcgccagttaatagtttgcgcaacgttgttgccatcgctacaggcatcgtggtgtcacgctcgtcgtttggtatggcttcattcagctccggttcccaacgatcaaggcgagttacatgatcccccatgttgtgcaaaaaagcggttagctccttcggtcctccgatcgttgtcagaagtaagttggccgcagtgttatcactcatggttatggcagcactgcataattctcttactgtcatgccatccgtaagatgcttttctgtgactggtgagtactcaaccaagtcattctgagaatagtgtatgcggcgaccgagttgctcttgcccggcgtcaatacgggataataccgcgccacatagcagaactttaaaagtgctcatcattggaaaacgttcttcggggcgaaaactctcaaggatcttaccgctgttgagatccagttcgatgtaacccactcgtgcacccaactgatcttcagcatcttttactttcaccagcgtttctgggtgagcaaaaacaggaaggcaaaatgccgcaaaaaagggaataagggcgacacggaaatgttgaatactcatattcttcctttttcaatattattgaagcatttatcagggttattgtctcatgagcggatacatatttgaatgtatttagaaaaataaacaaataggggtcagtgttacaaccaattaaccaattctgaacattatcgcgagcccatttatacctgaatatggctcataacaccccttgctcatgaccaaaatcccttaacgtgagttacgcgcgcgtcgttccactgagcgtcagaccccgtagaaaagatcaaaggatcttcttgagatcctttttttctgcgcgtaatctgctgcttgcaaacaaaaaaaccaccgctaccagcggtggtttgtttgccggatcaagagctaccaactctttttccgaaggtaactggcttcagcagagcgcagataccaaatactgttcttctagtgtagccgtagttagcccaccacttcaagaactctgtagcaccgcctacatacctcgctctgctaatcctgttaccagtggctgctgccagtggcgataagtcgtgtcttaccgggttggactcaagacgatagttaccggataaggcgcagcggtcgggctgaacggggggttcgtgcacacagcccagcttggagcgaacgacctacaccgaactgagatacctacagcgtgagctatgagaaagcgccacgcttcccgaagggagaaaggcggacaggtatccggtaagcggcagggtcggaacaggagagcgcacgagggagcttccagggggaaacgcctggtatctttatagtcctgtcgggtttcgccacctctgacttgagcgtcgatttttgtgatgctcgtcaggggggcggagcctatggaaaaacgccagcaacgcggcctttttacggttcctggccttttgctggccttttgctcacatgttctttcctgcgttatcccctgattctgtggataaccgtattaccgcctttgagtgagctgataccgctcgccgcagccgaacgaccgagcgcagcgagtcagtgagcgaggaagcggaaggcgagagtagggaactgccaggcatcaaactaagcagaaggcccctgacggatggcctttttgcgtttctacaaactctttctgtgttgtaaaacgacggccagtcttaagctcgggccccctgggcggttctgataacgagtaatcgttaatccgcaaataacgtaaaaacccgcttcggcgggtttttttatggggggagtttagggaaagagcatttgtcagaatatttaagggcgcctgtcactttgcttgatatatgagaattatttaaccttataaatgagaaaaaagcaacgcactttaaataagatacgttgctttttcgattgatgaacacctataattaaactattcatctattatttatgattttttgtatatacaatatttctagtttgttaaagagaattaagaaaataaatctcgaaaataataaagggaaaatcagtttttgatatcaaaattatacatgtcaacgataatacaaaatataatacaaactataagatgttatcagtatttattatcatttagaataaattttgtgtcgcccttaattgtgagcggataacaattacgagcttcatgcacagtggcgttgacattgattattgactagcatgttctttcctgcgttatcccctgattctgtggataaccgtattaccgccatgcattagttattaataacatacgctctccatcaaaacaaaacgaaacaaaacaaactagcaaaataggctgtccccagtgcaagtgcaggtgccagaacatttctct

**Supplementary file 4. Full plasmid sequence of the pGL-RF vector.** Different elements in the reporter plasmid were indicated using underline, square frame, and different colors of highlight and letters, respectively.
